# Trichiasis surgery: a patient-based approach

**Published:** 2010-12

**Authors:** Wondu Alemayehu, Amir Bedri Kello

**Affiliations:** Independent Consultant & General Manager, Berhan Public Health & Eye Care Consultancy. Email: **walemayehu@yahoo.com** or **walemayehu@berhan-health.org**; Senior Consultant, Light for the World. Email: **a.bedri@light-for-the-world.org** or **amirbedri@yahoo.com**

Trachoma is the leading infectious cause of blindness worldwide. Corneal scarring, which causes trachoma-related blindness, occurs when the upper eyelashes are turned inward and rub on the eye (cornea). This is called trichiasis, and if the lid margin turns inward, the term entropion is used. Currently, there are an estimated 8.2 million people with trichiasis and 3.1 million people are blind from trachoma.

A systematic review of population-based trachoma surveys has shown that women are affected by trichiasis approximately twice as often as men. Trichiasis is more common with increasing age; however, in communities with very high levels of trachoma infection, trichiasis can occasionally occur in children.

Persons who develop trachomatous trichiasis (TT) usually need treatment to either surgically turn the eyelashes outward from the eye or to remove one or two in-turning eyelashes which are not central or touching the cornea; the latter is pulling out the in-turned lash or lashes with forceps, a procedure called epilation. Bilamellar tarsal rotation (BLTR) or posterior lamellar tarsal rotation (PLTR) are procedures widely used in trachoma endemic countries to surgically treat TT and are believed to produce comparable results.

In some countries, there are huge numbers of persons with untreated TT, often living in poor and remote communities. For example, in Ethiopia, there are an estimated 1.2 million people with TT who need an operation. However, the number of TT operations currently performed each year in Ethiopia is about 80,000. At this rate, it will take 15 years just to clear the backlog, without considering any new cases which will occur!

There are several reasons for the large numbers of untreated trichiasis patients in endemic countries:

- Patients may be unaware that surgery can help, or they may be afraid of an eye operation; as a result, uptake of TT surgery is often low, even when surgery is provided free of charge. Sometimes, the fear is reinforced by awareness in the community that, at a particular clinic, trichiasis often comes back after surgery, which has a negative impact on uptake.
- Some patients find the cost of travel to seek eye services, or the lack of a companion to go with them, to be a significant obstacle. This is particularly true for women who also have to look after children and the household and cannot afford the time to go for treatment. Sometimes, it is just too great a distance to a health facility, so people will not go for treatment.
- In some situations, services are simply not available, nobody has been trained to perform trichiasis surgery, or the necessary equipment and consumables are not available.

**Figure:**
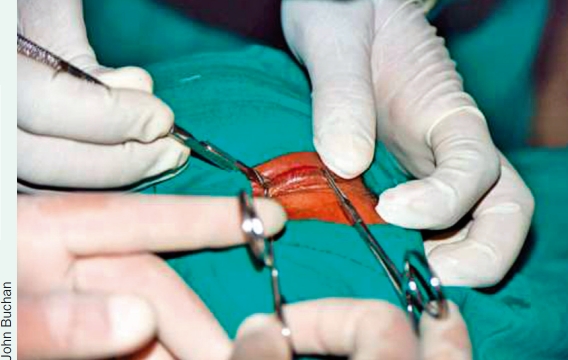
In some countries, there is a large backlog of trichiasis operations. NIGER

John Buchan

Strategies to address the TT backlog will vary from country to country and setting to setting.

These may include: creating awareness of treatment for trichiasis through health education, including radio programmes; ensuring TT surgery is available at low cost and close to where people with TT live; or conducting enhanced outreach in communities where trachoma is common.

TT surgery can be performed by well-trained ophthalmic nurses, assistants, or doctors. There is good evidence that TT surgery can be done by non-ophthalmologists with comparable results to those of ophthalmologists.

One of the challenges is to encourage the trained TT surgeon to continue to work in rural areas and to equip them so that they are able to perform sufficient TT operations per year to maintain good experience and quality.

Unfortunately, the quality of training of TT surgeons can be variable and adequate supervision may be lacking, leading to high rates of recurrence of TT after surgery. Surgeons who only do a small number of TT operations each month tend also to have poor surgical outcomes, leading to a vicious cycle of low uptake, low productivity, and poor surgical quality and outcome.

Breaking this cycle requires good planning and a willingness to acknowledge that results can be improved.

In order to develop a TT service it is useful to address various levels of eye care delivery.

## National level

- At a national level, it is necessary to identify areas with a high prevalence of TT and to prioritise these areas for TT surgery programmes.
- TT surgeons need to be given good quality training and be adequately equipped. Quality of care is essential. In order to improve the quality of surgery, training of TT surgeons should be standardised and surgeons should be certified using the World Health Organization manual on assessment of trichiasis surgeons.
- Due emphasis should be given to the selection of trainees, the creation of a career pathway, and supervision of TT surgeons.
- TT surgeons must also have adequate supplies of instruments and consumables.
- Services, whether static or outreach, that are staffed with poorly skilled, inadequately supervised TT surgeons can result in poor surgical outcomes and negative publicity for the programme.

## District level

- At the health centre level, transport to provide outreach programmes for TT surgery in affected communities is required, together with good provision of consumables to perform the operation: medicines, sutures, dressings, and so on.
- It may be necessary, in some situations, to consider offering incentives (such as a financial reward) to encourage good TT surgeons to work in high-volume TT programmes in remote areas.

## Community level

- At the community level, women must be specifically and deliberately targeted for trichiasis surgery. A successful trachoma programme requires the involvement of affected communities through their village leaders, women's group leaders, teachers, community health agents, health extension workers, or similar frontline health personnel. Recruiting village women who have had successful TT surgery to raise awareness and encourage others with TT to have the operation has proven to be a successful strategy.
- An outreach programme includes awareness creation in the community about trachoma, seeks community involvement in planning and executing the activities, and tries ultimately to engage the community to such an extent that they become the true owners of the programme. Community involvement and engagement is therefore essential for community ownership and the successful implementation of the full SAFE strategy.
- Community and local health service planners need to decide what contribution community members can make towards the cost of surgery. This will help to achieve sustainable service delivery and avoid a situation where the community either undervalues the service (perhaps because they consider a free service to be inferior) or where a state of dependence is created. Having said that, the cost of surgery should not be so high that poor patients cannot afford it.

In summary, it is essential to consider patients' needs. This will require comprehensive planning at national, district, and community levels to adapt and strengthen the health system to meet these needs. The ultimate aim is for patients with TT to have successful surgery, be satisfied with the result, and be advocates in their communities. Only then will we achieve the ultimate goal of the elimination of blinding trachoma.
